# Phenotypic heterogeneity within twins with MELAS with epilepsy: Case report

**DOI:** 10.1097/MD.0000000000047264

**Published:** 2026-05-01

**Authors:** Huiru Wu, Yanling Wang, Qingxia Kong

**Affiliations:** a Department of Neurology, Affiliated Hospital of Jining Medical University, Jining, China.

**Keywords:** epilepsy, gene mutation, m.3243A>G, MELAS, treatment, twins

## Abstract

**Rationale:**

Mitochondrial encephalomyopathy with lactic acidemia and stroke-like episodes (MELAS) syndrome is a maternally inherited mitochondrial disorder caused by mutations in mitochondrial DNA, most commonly the m.3243A>G variant. This mutation impairs oxidative phosphorylation, leading to inadequate cellular energy production, particularly in high-demand tissues such as the brain and muscles. The resultant energy deficit manifests as neurological and muscular dysfunction, including stroke-like episodes, seizures, and lactic acidosis.

**Patient concerns:**

Twin brothers presented with heterogeneous clinical characteristics. The elder twin experienced seizures, blurred vision, hypertrichosis, exercise intolerance, and had learning difficulties since age 10. The younger twin developed hearing loss at age 12, followed by persistent epileptic seizures 3 months later. Both had a history of progressive neurological and multisystemic symptoms suggestive of a metabolic disorder.

**Diagnoses:**

Diagnostic evaluations included electroencephalography (EEG), which showed widespread mixed high-amplitude slow waves, and cranial magnetic resonance imaging, which revealed migratory lesions that changed with recurrent episodes. Genetic testing confirmed the m.3243A>G mutation in both twins. Their mother was identified as an asymptomatic carrier with an estimated heteroplasmy level of 30.79%.

**Interventions:**

The elder twin was initially treated with acyclovir (antiviral) and methylprednisolone (anti-inflammatory) for suspected viral encephalitis, with symptomatic support. After genetic confirmation of MELAS, supportive therapies included coenzyme Q10, adenosine triphosphate disodium, levocarnitine, and arginine. During recurrent admissions for status epilepticus, antiepileptic regimens were maintained or adjusted, and imaging (magnetic resonance imaging/electroencephalogram) was repeatedly used for monitoring. His brother received similar interventions – levetiracetam, coenzyme Q10, and adenosine triphosphate disodium – upon diagnosis, with additional management for seizures, headaches, and gastrointestinal symptoms.

**Outcomes:**

Both twins were definitively diagnosed with MELAS syndrome. The elder twin was diagnosed first based on clinical and genetic findings, while the younger twin was diagnosed after the emergence of hearing loss and seizures. The condition highlights the progressive and variable nature of MELAS.

**Lessons:**

The case underscores the significant phenotypic heterogeneity of MELAS, which often leads to misdiagnosis or delayed diagnosis. Early genetic testing is critical for accurate identification and prompt intervention. Family screening is recommended due to the maternal inheritance pattern, and tailored management should address the multifaceted clinical manifestations.

## 1. Introduction

Mitochondrial encephalomyopathy with lactic acidosis and stroke-like episodes (MELAS) syndrome is a common maternally inherited mitochondrial diseases.^[1]^ MELAS is usually associated with the 3243A>G mutation located in the mitochondrial transfer RNA^Leu (UUR)^ gene (MT-TL-1). Mutations at this site result in impaired mitochondrial translation and protein synthesis and diminished mitochondrial adenosine triphosphate (ATP) production.^[2,3]^ As the specificity of the clinical manifestations of MELAS is low, diagnosis is difficult. Here, we report on identical twin brothers with MELAS (m.3243A>G gene mutation), where the onset of disease was accompanied by different clinical presentations. We describe the electrophysiology, imaging, genetic testing, and diagnostic and therapeutic procedures for these patients. The patients gave informed consent to publish their clinical data. We present the following case in accordance with the CARE reporting checklist.

## 2. Case presentation

### 2.1. Clinical information

On October 25, 2020, a 10-year-old boy, the eldest identical twin of 2 males born on March 3, 2010, was admitted to the intensive care unit of our hospital with sudden onset but recurrent unconsciousness, twitching of limbs, and blurred vision during the night. The patient did not experience foaming at the mouth or urinary or fecal incontinence. On admission, physical examination showed emaciation and normal development. Laboratory tests showed creatine kinase-myocardial band, 13.62 ng/mL and normal liver and kidney function, electrolytes, and fasting blood glucose. Lumbar puncture showed the cerebrospinal fluid was colorless and transparent, and cerebrospinal fluid smear and culture were not abnormal. Cerebrospinal fluid analysis showed glucose levels were >2.75 mmol/L and chloride levels were normal. Tests for autoimmune brain, demyelination, and oligoclonal protein were negative. Cranial magnetic resonance imaging (MRI; October 27, 2020) showed no significant abnormalities. Electroencephalogram (EEG) showed the presence of multiple widespread medium–high amplitude 1.5 to 4 HZ mixed slow waves interspersed with low–moderate amplitude fast waves continuously discharged during waking hours. Repeat cranial MRI (November 07, 2020) showed an abnormal signal in the left temporoparieto-occipital region, a slightly heightened T2-weighted-fluid-attenuated inversion recovery signal, a restricted diffusion pattern on diffusion weighted imaging (DWI), and a low apparent diffusion coefficient value. Viral encephalitis was considered, and the patient was given acyclovir (antiviral), methylprednisolone (anti-inflammatory) and other symptomatic treatments, and his condition improved. After treatment, there was no recurrence of limb convulsions or confusion, and the patient had normal vision in both eyes. He was discharged from the hospital with methylprednisolone and vitamin B12.

### 2.2. Diagnosis and treatment

**Figure 1.**
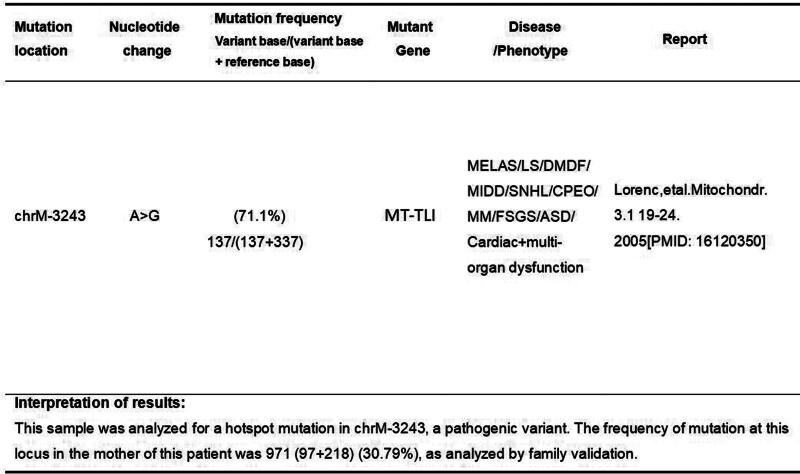
The patient underwent mitochondrial full genome analysis. MELAS = mitochondrial encephalomyopathy with lactic acidemia and stroke-like episodes, MT-TL1 = mitochondrial transfer RNA^Leu (UUR)^ gene.

In July 2021, the patient was admitted to our hospital with persistent status epilepticus. The MRI protocol for epilepsy showed no significant abnormalities (July 15, 2021). Electroencephalogram showed multiple sharp waves and sharp-slow complex waves on the left side of the brain during all phases of waking and sleeping. On Raven’s Progressive Matrices, the patient scored 25 correct questions, his IQ was 62, and he had a mildly imbecilic level of intelligence. The patient’s parents were both healthy and the marriage was non-consanguineous. There was no family history of similar disease. The patient’s mother was healthy during pregnancy. At school, the patient’s academic performance was poor. During extracurricular activities, the patient felt fatigued, and he often had indigestion and vomiting. Taking into account the patient’s medical history, physical examination and ancillary test results, the possibility of genetic mitochondrial disease was considered, and genetic testing was recommended. Results suggested the presence of the mtDNA 3243A>G mutation (Fig. 1). Genetic testing showed the estimated mutation rate at this locus in his mother was 30.79%. Finally, the patient was diagnosed with MELAS. He was treated for MELAS with lacosamide, levetiracetam tablets, and leucovorin oral solution. The patient’s brother was normal at the time and had no clinical symptoms, so he did not undergo genetic testing.

**Figure 2.**
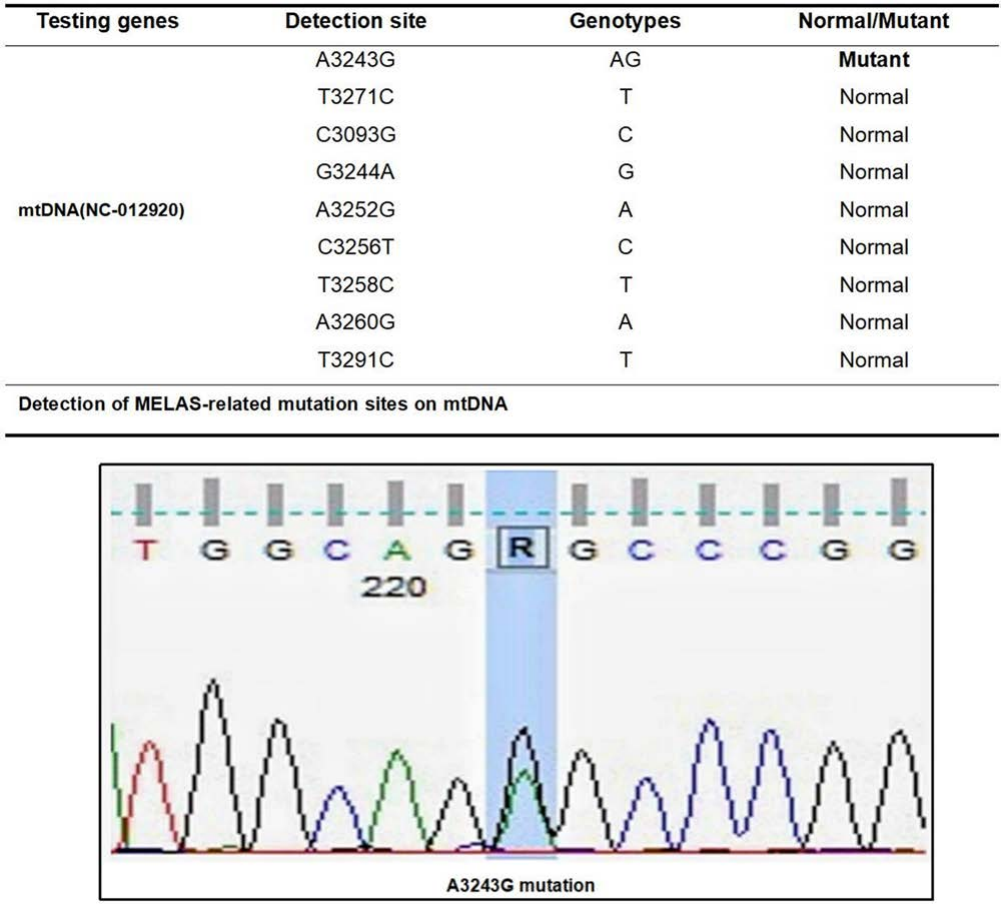
Genetic testing of the patient’s brother suggested mutation loci for MELAS. MELAS = mitochondrial encephalomyopathy with lactic acidemia and stroke-like episodes.

In August 2022, the patient’s brother presented to our hospital with sudden binaural hearing loss and tinnitus, with no other symptoms. Physical examination showed a long thin body and long hair on the skin of the extremities. Considering that MELAS can present similar clinical manifestations, genetic testing was completed, and the results suggested the presence of the mtDNA A3243A>G mutation (Fig. 2). In November 2022, the patient’s brother was admitted to our hospital with a history of seizure 3 months earlier and sudden-onset status epilepticus. There was no obvious abnormality on cranial MRI (November 18, 2022). He was given levetiracetam tablets, adenosine triphosphate disodium tablets, and coenzyme Q10.

### 2.3. Follow-up

**Figure 3.**
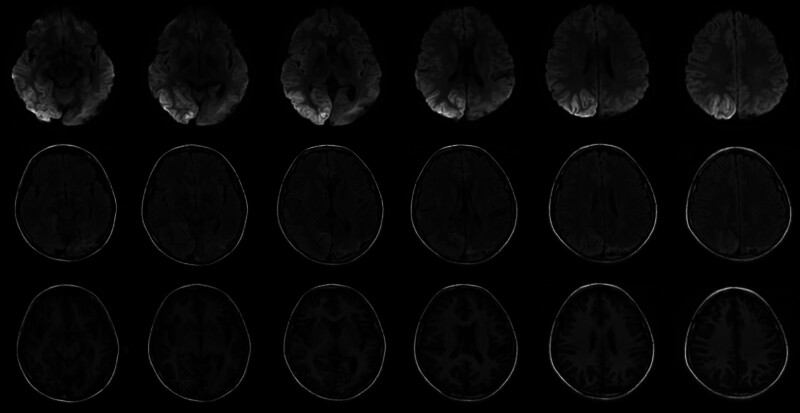
The patient underwent a cranial MRI on October 26, 2022. MRI = magnetic resonance imaging.

The patient was readmitted to our hospital 1 year after the original diagnosis of MELAS with difficulty opening his eyes, and persistent status epilepticus. Cranial MRI (October 26, 2022) showed regions with long T1-long T2 signals and a high signal on DWI in the right temporoparieto-occipital lobe and a mildly elevated signal on DWI in the left temporoparieto-occipital lobe (Fig. 3). The patient was prescribed lacosamide, levetiracetam, levocarnitine, coenzyme Q10, and arginine. Cranial MRI (February 02, 2023) showed large regions of long T1 and T2 signals in the bilateral temporoparieto-occipital lobes, and a high signal on DWI in the left temporoparietal lobe. The patient continues to have intermittent tonic clonic seizures, mostly sustained, and is currently blind.

**Figure 4.**
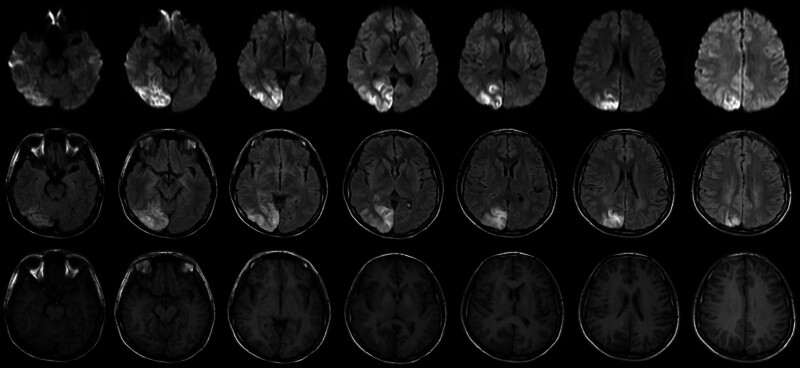
The patient’s brother underwent a cranial MRI on January 14, 2024. MRI = magnetic resonance imaging.

The patient’s brother was discharged from the hospital with intermittent convulsive seizures, sometimes status epilepticus, recurrent headaches and blurred vision. Cranial MRI (January 14, 2024) showed a large region with a long T2 signal and a high signal on DWI in the right occipital lobe (Fig. 4). In August 2024, the patient’s brother was hospitalized for persistent status epilepticus, and cranial MRI showed a large region with a low signal on T1WI, a high signal on T2WI, and a localized high signal on DWI in the left temporal-parieto-occipital lobe. The patient’s brother was treated for recurrent headaches and nausea and vomiting and he experienced no further seizures during hospitalization. His visual field deficits were resolved.

## 3. Discussion

Accumulating evidence suggests that MELAS is caused by point mutations on mtDNA. At least 10 locus-related mutations in mtDNA (e.g., 3243, 3271, 3260, 3252, 1642) have been reported, among which 3243tRNALeu (UUR) A→G (A3243G) is the most common, occurring in approximately 80% of patients with MELAS.^[4,5]^ As mature sperm do not carry mitochondrial DNA, most patients with MELAS inherit altered mitochondrial DNA from their mother. This report describes identical male twins with MELAS whose mother carried the causative gene.

One twin brother was admitted to our hospital with sudden onset but recurrent unconsciousness, twitching of limbs, and blurred vision during the night. He was misdiagnosed as viral encephalitis, mainly due to the similarity of clinical manifestations between MELAS and viral encephalitis, including headache, convulsions, slowing of the background dominant rhythm on EEG, and abnormal signals in the occipital lobe on cranial MRI. The patient underwent multiple cranial MRIs at different times, which showed changes with progression and regression of his disease that were not associated with regional blood flow, with some foci improving or disappearing, and new foci appearing. We recommend the possibility of MELAS should be considered when clinical encounters are characterized by a typical presentation, slowing of EEG activity, and cranial MRI showing foci located in cortical and subcortical white matter and multifocal, asymmetric and changing signal abnormalities not associated with regional blood flow.

The twin brothers had epileptic seizures, which were considered manifestations of mitochondrial disease. Mitochondrial dysfunction leading to ATP depletion results in the occurrence of seizures in mitochondrial diseases. Impaired membrane channels and Na^+^/K^+^ ATPase activity lead to loss of neuronal hyperpolarization,^[6,7]^ while defects in mitochondrial metabolism cause cells to become overexcited through several mechanisms: death of inhibitory interneurons (especially in the occipital cortex) leading to a decrease in GABA-mediated inhibition; suppression of inhibitory neuronal activity in hippocampal interneurons; and an increase in glutamate release from the synapses of astrocytes.^[8-10]^ Hearing loss and tinnitus are less specific clinical manifestations of MELAS. Some studies show that 27% to 75% of patients with MELAS experience hearing loss, mostly sensorineural hearing loss that is characterized by gradual onset, gradual progression and partial recovery.^[11]^ Based on these observations, we recommend careful physical examination, detailed medical and family history, and a combination of laboratory examination, electrophysiology, imaging, and genetic testing in patients with a suspected diagnosis of MELAS.

In this case, both twins were diagnosed with MELAS, but the pharmacological treatment was ineffective. There is no cure for MELAS, and most strategies are aimed at managing symptoms. Levetiracetam in combination with benzodiazepines (e.g., clonazepam or clobazole) is considered a safe and effective treatment for mitochondrial disease with seizures. Zonisamide may be considered for patients with MELAS or drug-resistant epilepsy, and lacosamide is a good adjunct therapy. Lamotrigine may exacerbate myoclonic seizures.^[12-15]^

In vitro studies show mitochondrial activity of MELAS patient cells are restored through activation of mutant tRNAs,^[16]^ and mitochondrial transfer from highly purified mesenchymal stem cells (RECs) can improve the function of neurons derived from organisms with MELAS.^[17]^ However, these strategies have not been implemented clinically.

## 4. Conclusions

In conclusion, we report twin brothers carrying the mitochondrial 3243 A>G mutation, both of whom were diagnosed with MELAS, but who had different clinical manifestations. We recommend genetic screening and genetic counseling for families with a high suspicion of MELAS, to alleviate the burden of this genetic disorder on families and society. This report should increase clinical awareness and early diagnosis of MELAS.

## Author contributions

**Conceptualization**: Huiru Wu.

**Data curation**: Yanling Wang.

**Investigation**: Yanling Wang.

**Writing – original draft**: Huiru Wu.

**Writing – review & editing**: Qingxia Kong.
